# Spatiotemporal epidemiology, environmental correlates, and demography of malaria in Tak Province, Thailand (2012–2015)

**DOI:** 10.1186/s12936-019-2871-2

**Published:** 2019-07-16

**Authors:** Chris Erwin G. Mercado, Saranath Lawpoolsri, Prayuth Sudathip, Jaranit Kaewkungwal, Amnat Khamsiriwatchara, Wirichada Pan-ngum, Surapon Yimsamran, Siam Lawawirojwong, Kevin Ho, Nattwut Ekapirat, Rapeephan R. Maude, Jacher Wiladphaingern, Verena I. Carrara, Nicholas P. J. Day, Arjen M. Dondorp, Richard J. Maude

**Affiliations:** 1 Mahidol Oxford Tropical Medicine Research Unit (MORU), Faculty of Tropical Medicine, Mahidol University, Bangkok, Thailand; 2 Department of Tropical Hygiene, Faculty of Tropical Medicine, Mahidol University, Bangkok, Thailand; 3 Center of Excellence for Biomedical and Public Health Informatics (BIOPHICS), Faculty of Tropical Medicine, Mahidol University, Bangkok, Thailand; 4 Bureau of Vector-borne Diseases (BVBD), Department of Disease Control (DDC), Ministry of Public Health (MOPH), Nonthaburi, Thailand; 5 Geo-Informatics and Space Technology Development Agency (GISTDA), Bangkok, Thailand; 6 Shoklo Malaria Research Unit (SMRU), Faculty of Tropical Medicine, Mahidol University, Mae Sot, Tak Thailand; 7 Centre for Tropical Medicine and Global Health, Nuffield Department of Medicine, University of Oxford, Oxford, UK; 8 Department of Epidemiology, Harvard T.H. Chan School of Public Health, Harvard University, Boston, USA

**Keywords:** Malaria, Surveillance, Climate, Forest, Risk mapping, Epidemiology, Thailand

## Abstract

**Background:**

Tak Province, at the Thai–Myanmar border, is one of three high malaria incidence areas in Thailand. This study aimed to describe and identify possible factors driving the spatiotemporal trends of disease incidence from 2012 to 2015.

**Methods:**

Climate variables and forest cover were correlated with malaria incidence using Pearson’s *r*. Statistically significant clusters of high (hot spots) and low (cold spots) annual parasite incidence per 1000 population (API) were identified using Getis-Ord Gi* statistic.

**Results:**

The total number of confirmed cases declined by 76% from 2012 to 2015 (*Plasmodium falciparum* by 81%, *Plasmodium vivax* by 73%). Incidence was highly seasonal with two main annual peaks. Most cases were male (62.75%), ≥ 15 years (56.07%), and of Myanmar (56.64%) or Thai (39.25%) nationality. Median temperature (1- and 2-month lags), average temperature (1- and 2-month lags) and average relative humidity (2- and 3-month lags) correlated positively with monthly total, *P. falciparum* and *P. vivax* API. Total rainfall in the same month correlated with API for total cases and *P. vivax* but not *P. falciparum*. At sub-district level, percentage forest cover had a low positive correlation with *P. falciparum*, *P. vivax*, and total API in most years. There was a decrease in API in most sub-districts for both *P. falciparum* and *P. vivax*. Sub-districts with the highest API were in the Tha Song Yang and Umphang Districts along the Thai–Myanmar border. Annual hot spots were mostly in the extreme north and south of the province.

**Conclusions:**

There has been a large decline in reported clinical malaria from 2012 to 2015 in Tak Province. API was correlated with monthly climate and annual forest cover but these did not account for the trends over time. Ongoing elimination interventions on one or both sides of the border are more likely to have been the cause but it was not possible to assess this due to a lack of suitable data. Two main hot spot areas were identified that could be targeted for intensified elimination activities.

**Electronic supplementary material:**

The online version of this article (10.1186/s12936-019-2871-2) contains supplementary material, which is available to authorized users.

## Background

There is a goal to eliminate malaria across countries in the Greater Mekong Subregion (Cambodia, Yunnan Province in China, Lao People’s Democratic Republic (PDR), Myanmar, Thailand and Viet Nam) by 2030. In Thailand, malaria remains as a major public health priority with over 13 million people (19% of the total population) currently at risk [1], most of whom are situated along border areas with its neighbouring countries [2]. There is significant geographical heterogeneity in spatial distribution of disease incidence, which exemplifies the “border malaria” type [3], characterized by high transmission along international borders [3–5].

Thailand has made significant progress towards its goal of eliminating malaria by 2024 [2]. Between 2012 and 2017, Thailand reduced its malaria cases by 67%, with an accelerated decrease of 39% during the period 2016 to 2017 [6] with scale-up of malaria elimination interventions. According to the 2018 World Malaria Report, the country had 11,440 reported confirmed cases in health facilities (which include 1075 cases at community level and 3023 cases from the private sector) and 11 reported deaths due to malaria in 2017. With evidence of emerging artemisinin drug resistance in *Plasmodium falciparum* malaria in the Thailand–Myanmar and Thailand–Cambodia–Lao PDR border areas [7–12], including insecticide resistance [13], adding complexity to the elimination agenda in Thailand, it is increasingly important to understand the local epidemiology of malaria to guide targeted elimination efforts. Strengthening malaria surveillance has been highlighted by the World Health Organization as a core intervention to accelerate progress towards elimination [14, 15].

Tak Province, at the Thai–Myanmar border, is one of three high incidence areas in Thailand. It has historically recorded one of the highest incidences of malaria cases across the country [16]. The province reported the highest number of malaria cases nationwide in 2012 and 2013 [17], and ranked second only to Ubon Ratchathani Province the following 2 years [18]. In 2015, Tak accounted for 21% of total reported cases in Thailand [19]. *Plasmodium vivax* is the predominant species, reflective of the national situation [1]. The availability of detailed climate and forest cover data presents an opportunity to better understand the epidemiology of malaria in the area.

This study analysed routine malaria surveillance data from 2012 to 2015 to describe and identify possible factors driving the spatiotemporal trends of disease incidence.

## Methods

### Study setting

**Fig. 1:**
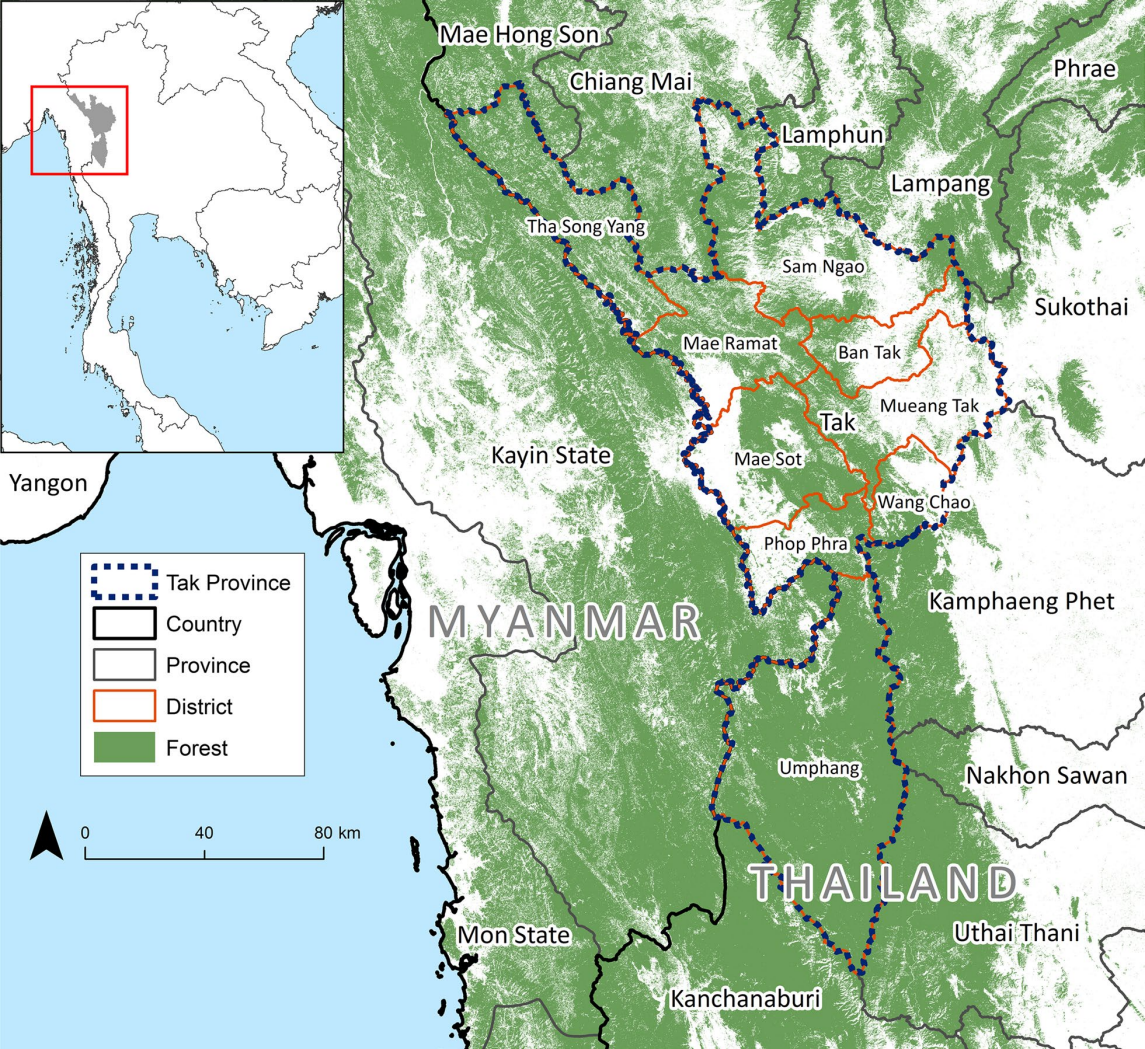
Reference map. Place names are provided for the nine districts (*amphoe*) subdividing the province

The study location was Tak Province in western Thailand. It is subdivided into nine administrative districts (Fig. 1), 60 sub-districts [20], and 493 villages, with a total population of 526,381 as of the 2010 national census [21]. The climate in Tak is characterized as tropical with rainy season from May to October [22]. The province has a total land area of 16,406.6 km^2^ (fourth largest among 76 Thai provinces). At its western edge, Tak Province shares international borders with Kayin State of Myanmar.

### Malaria diagnosis

In this area, malaria is diagnosed mostly through passive case detection by light microscopy by trained microscopists at malaria posts, health promoting hospitals and district hospitals. This is supplemented by active case detection (specifically by reactive case detection) [23]. Rapid diagnostic test (RDT) (SD BIOLINE Malaria Ag Pf/Pv^®^) are also used. There is also a system of village volunteer health workers who facilitate access to malaria diagnosis and treatment. Data on reported malaria cases is thought to be of high completeness and quality [24].

### Data collection

Information about numbers of confirmed malaria cases at sub-district level, between 1 January 2012 and 31 December 2015 were extracted from the consolidated malaria database of the Department of Disease Control, Ministry of Public Health (MOPH). This included individual data gathered from both the Bureau of vector-borne diseases (BVBD) and Bureau of epidemiology (BOE) national malaria surveillance systems. These two databases have been combined since 2012 with removal of duplicate cases from these two sources through a collaborative project supported by the Global Fund [19, 24, 25]. A confirmed case was any person with a positive malaria blood smear or RDT result reported by government health workers [2]. Data included age, sex, and nationality (nationals of Thailand = “Thai”, nationals of Myanmar = “Myanmar”, nationals of Lao PDR = “Lao” and “Other”), as well as address to sub-district level, date of diagnosis and parasite species.

Aside from malaria incidence data, other information used in this study was (i) annual population estimates from the Ministry of Interior (http://www.moi.go.th), (ii) administrative boundary layers for Thailand from the Ministry of Interior (http://www.moi.go.th), with the most recent available version being from 2015; (iii) weather station data, collected as daily measurements of rainfall (in millimetres), temperature (in degrees Celsius,  °C) and relative humidity (in percentage), from the Thai Meteorological Department (https://www.tmd.go.th); (iv) forest cover layers from combining publicly available rasters characterizing global forest extent and change from 2000 through 2016 derived from Landsat data (http://earthenginepartners.appspot.com/science-2013-global-forest) [26]. These environmental variables were chosen as they are what was available from the climate monitoring stations in Thailand and previous studies had found correlation between each of them and malaria [27–29]. Forest cover was used as much of the malaria transmission in Thailand and the broader Greater Mekong Subregion is known to be in forest and forest fringe areas [30]. Spatial data management and processing were done in ArcCatalog 10.3.1 (Esri, Redlands, California, USA) and Excel 2013 (Microsoft, Redmond, Washington, USA).

### Data analysis

Spatial and temporal trends in malaria incidence were examined at sub-district level. Thematic maps were produced using ArcMap 10.3.1 (Esri, Redlands, California, USA). Mixed infections were added to numbers of both *P. falciparum*-only and *P. vivax*-only cases prior to mapping of numbers of confirmed cases and annual parasite incidence (API) by species. API was calculated as the number of confirmed cases per 1000 total population as estimates of population at risk in each sub-district were not available. *Plasmodium falciparum*-to-*P. vivax* ratio was the number of confirmed *P. falciparum* divided by the number of confirmed *P. vivax* monoinfections. Hotspot maps [31] of API were produced using the Hot Spot Analysis (Getis-Ord Gi*) tool in ArcMap. Spatial relationships among features (i.e. sub-district boundaries) were defined by fixed distance band, and calculated with Euclidean (straight-line) distance. Correlations between malaria incidence rate, climate measurements and percent forest cover were examined using Pearson’s *r* in GraphPad Prism version 7.04 for Windows (GraphPad Software, La Jolla, California, USA). Age-sex distributions of malaria cases were plotted using R version 3.4.4 (The R Foundation for Statistical Computing, Vienna, Austria). Time series analysis of monthly malaria cases was done using stl() function (“seasonal and trend decomposition using LOESS) [32], also in R.

Forest cover was calculated by combining annual rasters of forest change and baseline forest cover in 2012 to derive annual forest cover layers at 30 m resolution. These were then combined with sub-district boundary files to calculate percentage forest cover by sub-district. Change in forest cover from 2012 to 2015 was calculated directly from the annual rasters. This was correlated with change in API using Pearson’s correlation on the logged values. Daily climate data from the five climate stations in Tak Province were averaged and then summarized by month as mean, median, maximum and minimum values. These were then correlated with monthly API at province level using Pearson’s correlation.

### Ethical considerations

Permission to access consolidated and de-identified malaria surveillance data was provided by the MOPH. The protocol for this study was reviewed and approved by the Faculty of Tropical Medicine Ethics Committee (FTM-EC), Mahidol University.

## Results

### Total cases

**Fig. 2:**
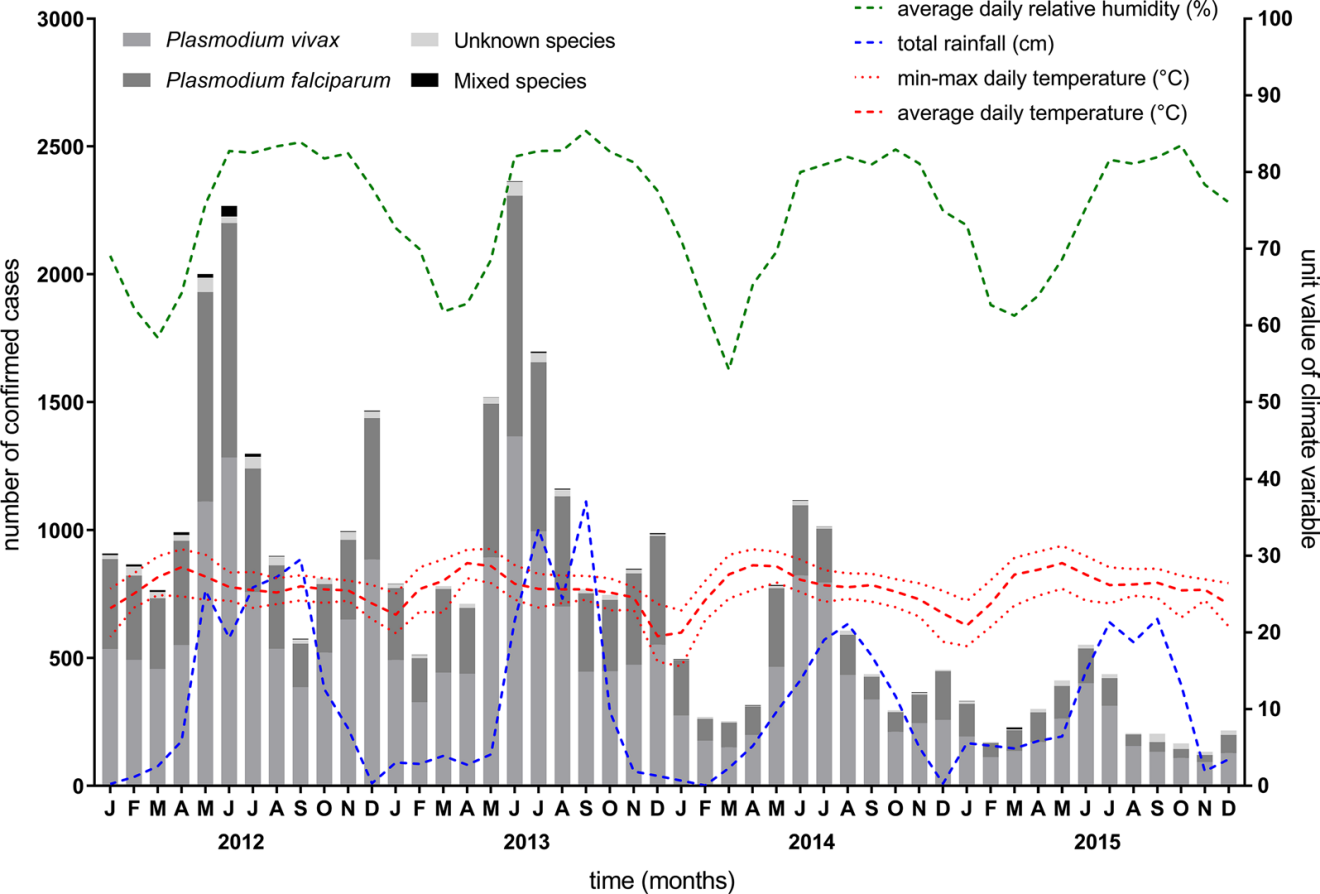
Monthly malaria cases and climate station measurements in Tak Province, 2012–2015. Each bar represents the total numbers of confirmed malaria cases in the province, including those with unknown species, from January 2012 to December 2015. Coloured lines represent corresponding monthly climate measurements (relative humidity; total rainfall; minimum, maximum, and average temperature)

From 2012 to 2015, there were 36,536 confirmed malaria cases in Tak Province (Fig. 2 and Table 1). In each surveillance year, the majority of cases were infected with *P. vivax*. On average, these two species combined comprised at least 97% of total cases annually with only 878 cases reported as unknown species (an overall percentage of 2.40% over the four years).

**Table 1:** Confirmed malaria cases by type of species in Tak Province, 2012–2015

Year	*P. falciparum*		*P. vivax*		*P. malariae*		Mixed		Unknown		Total
	N	%	N	%	N	%	N	%	N	%	N
2012	5215	37.62	8165	58.90	18	0.13	112	0.81	353	2.55	13,863
2013	5039	39.05	7575	58.71	14	0.11	22	0.17	253	1.96	12,903
2014	1926	30.04	4369	68.14	6	0.09	10	0.16	101	1.58	6412
2015	967	28.80	2207	65.72	5	0.15	8	0.24	171	5.09	3358
Total	13,147	35.98	22,316	61.08	43	0.12	152	0.42	878	2.40	36,536

“Mixed” refers to Pf + Pv

The general population of Tak Province represented almost 1% of the national total during the study period. Total API in Tak has steadily declined from 2012 to 2015 by 73.93%, with a marked decrease of at least 50% from 2013 to 2014 and again from 2014 to 2015. Similar downward trends were seen for *P. falciparum* and *P. vivax* infections. *Plasmodium falciparum*-to-*P. vivax* ratio decreased from 0.64 in 2012 to 0.44 in 2015. A summary of annual API by species and *P. falciparum*-to-*P. vivax* ratio is shown in Table 2.

**Table 2:** Population, annual parasite incidence and Pf:Pv ratio in Tak Province, 2012–2015

Year	Population	API			Pf:Pv ratio
		Total cases	Pf + mixed	Pv + mixed	
2012	526,045	26.35	10.13	15.73	0.64
2013	532,353	24.24	9.51	14.27	0.67
2014	539,553	11.88	3.59	8.12	0.44
2015	618,382	5.43	1.58	3.58	0.44

Annual population figures were based on civil registration records that comprised both Thai and foreign nationals

### Exploration of seasonal trend

**Fig. 3:**
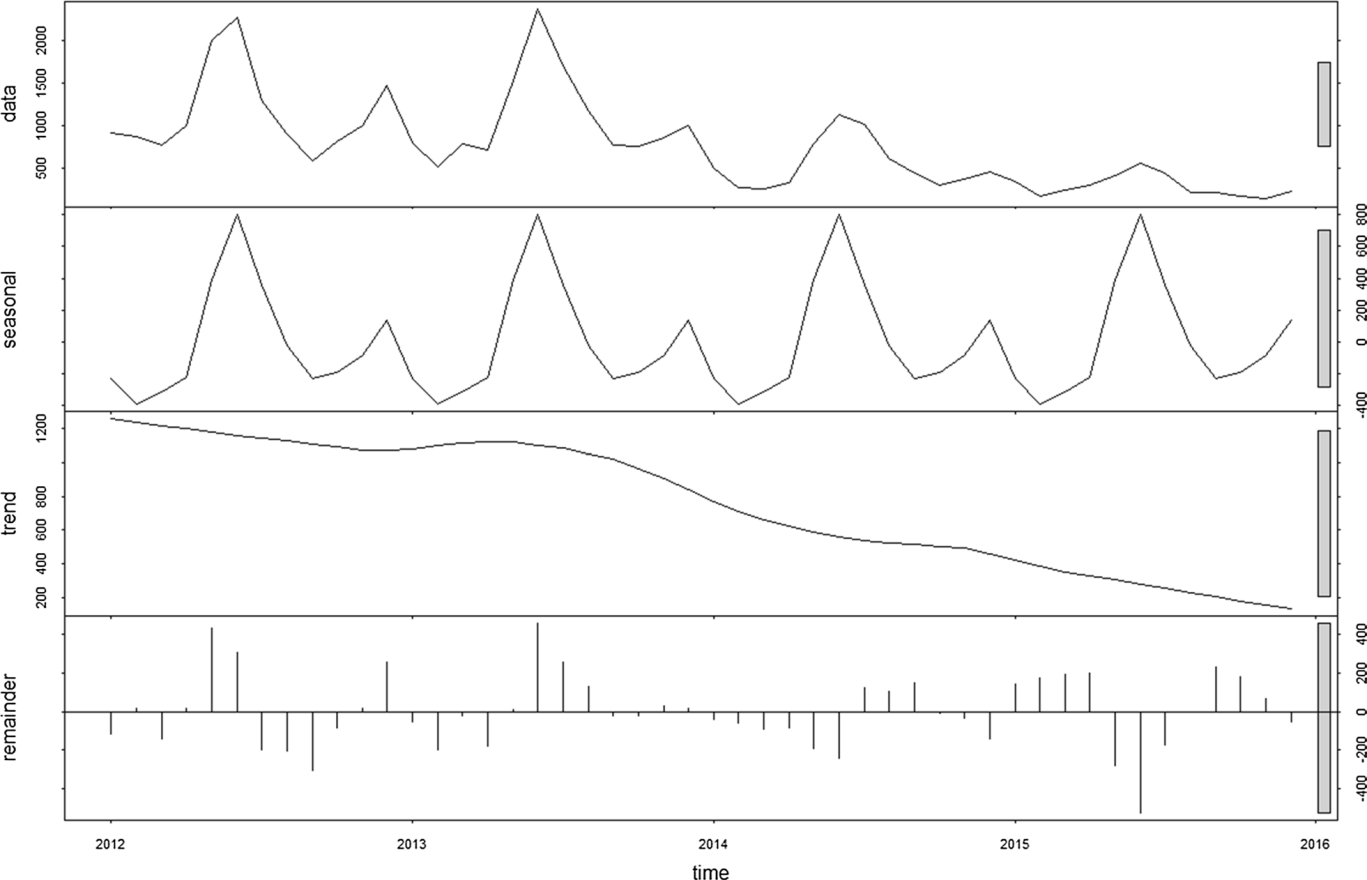
Time series decomposition of total confirmed malaria cases in Tak Province, 2012–2015. Top plot is decomposed into seasonal, trend and irregular components. The grey bar on the right indicates the relative magnitude of each of the decomposed components (length of bar = relative magnitude)

**Fig. 4:**
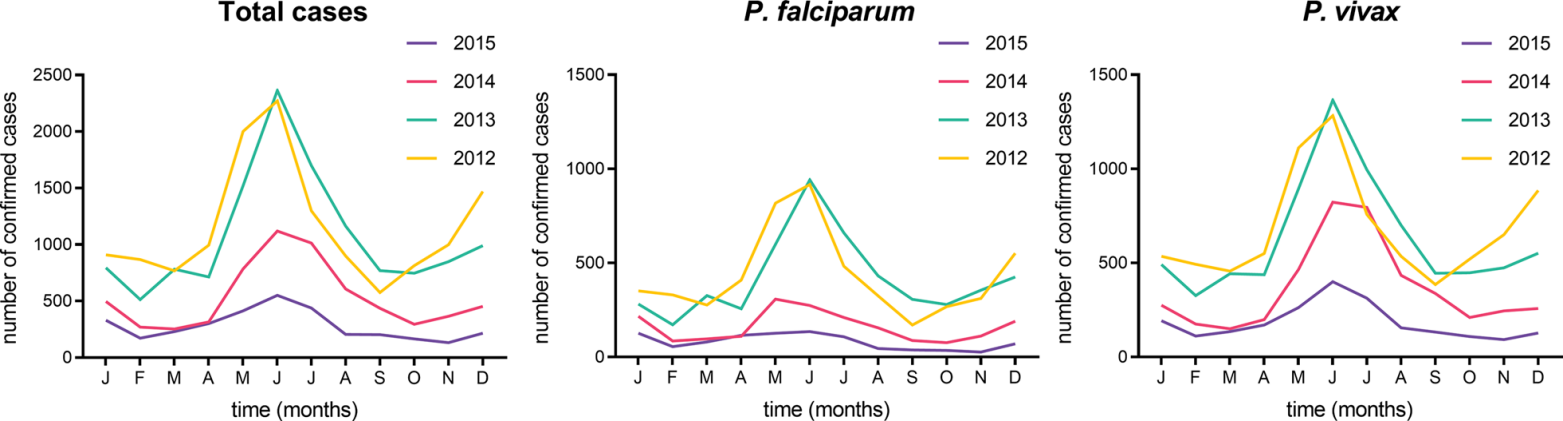
Confirmed malaria cases by month in Tak Province, 2012–2015. *Plasmodium falciparum* and *P. vivax* cases shown are numbers of mono-infections

**Fig. 5:**
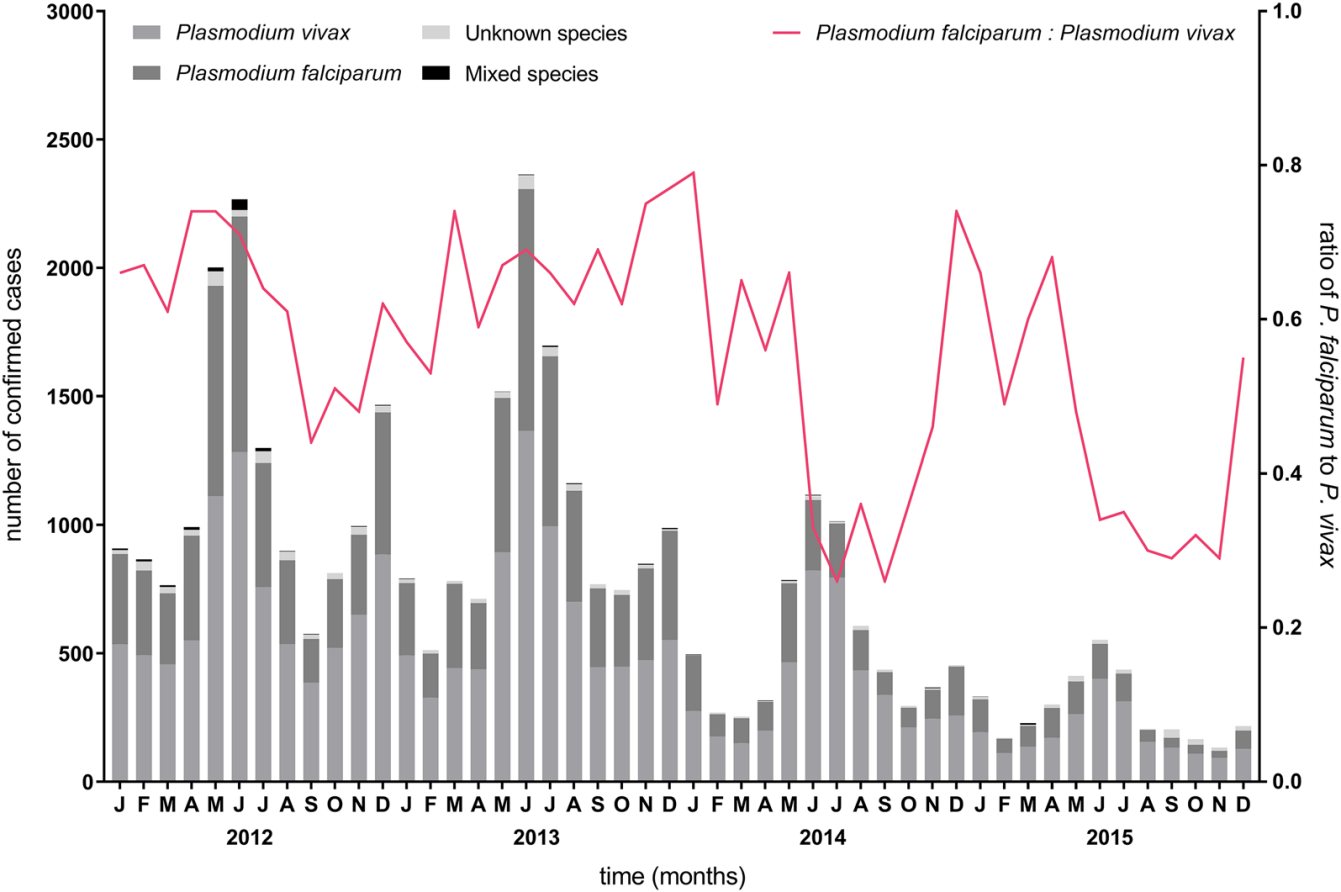
*Plasmodium falciparum*-to-*P. vivax* ratio (mono-infections) by month in Tak Province, 2012–2015

The time series decomposition of malaria cases in Tak Province shows a seasonal pattern and a declining trend from January 2012 to December 2015 (Fig. 3) with the same pattern being seen for each of *P. falciparum* (Additional file 1: Fig. S1) and *P. vivax* (Additional file 1: Fig. S2). The peak number of total cases occurred in the month of June, a seasonal trend similarly seen in both major malaria species (Fig. 4). *P. falciparum*-to-*P. vivax* ratio by month is shown in Fig. 5.

### Patient demographics

Demographic characteristics of people with malaria for the province are summarized by year in Table 3. Most cases were male (n = 22,926, 62.75%), ≥ 15 years (56.07%), and Myanmar (56.64%) or Thai (39.25%) nationality. Only 86.12% (n = 31,464) of total cases had their age recorded [all from BVBD, with four outliers (age > 150)].

**Table 3:** Demographic profile of malaria cases in Tak Province, 2012–2015

Variable	2012		2013		2014		2015		Total	
	N	%	N	%	N	%	N	%	N	%
Sex										
Male	8690	62.68	8053	62.41	4053	63.21	2130	63.43	22,926	62.75
Female	5173	37.32	4850	37.59	2359	36.79	1228	36.57	13,610	37.25
Age group (in years)										
0 to 14	5290	44.74	4940	44.20	2463	43.21	1129	40.86	13,822	43.93
15 to 64	6391	54.05	6096	54.55	3159	55.42	1601	57.94	17,247	54.82
Over 64	144	1.22	140	1.25	78	1.37	33	1.19	395	1.26
Nationality										
Myanmar	7593	54.77	7582	58.76	3645	56.85	1875	55.84	20,695	56.64
Thai	5654	40.78	4851	37.60	2532	39.49	1303	38.80	14,340	39.25
Other	606	4.37	470	3.64	233	3.63	180	5.36	1489	4.08
Lao	10	0.07	0	0.00	2	0.03	0	0.00	12	0.03

Column percentages are presented

**Fig. 6:**
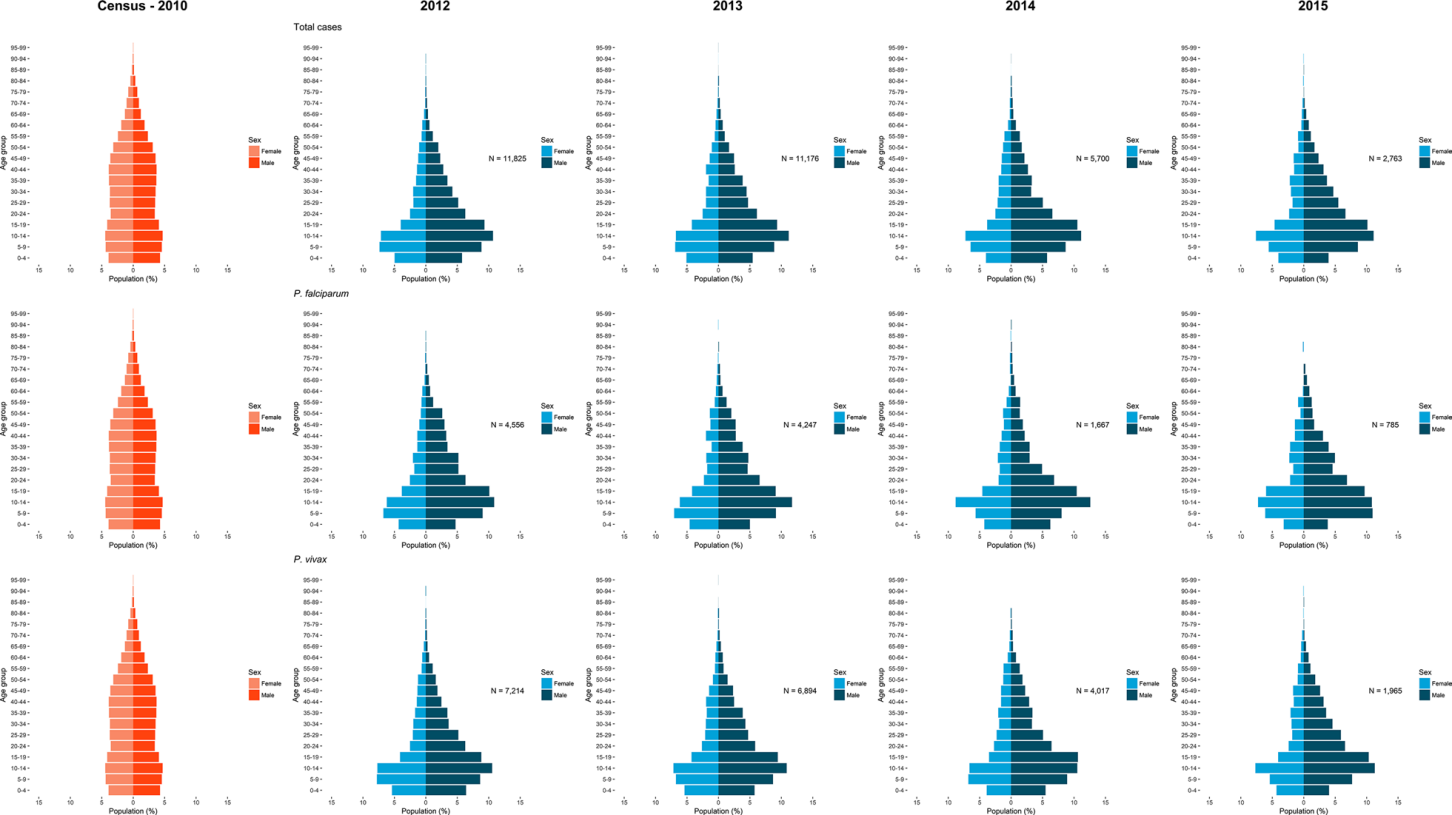
Age-sex distributions (pyramid) of malaria cases in Tak Province, 2012–2015. First column pyramids represent the population in Tak Province as of the most recent census in 2010 (N = 526,376). The second to fifth columns show the corresponding annual patterns of confirmed malaria cases, where the rows represent type of infection (top: Total cases, middle: *P. falciparum* mono-infections, bottom: *P. vivax* monoinfections)

The age-sex distributions of malaria cases are presented as annual population pyramids in Fig. 6. These are compared to the provincial population age-sex structure as of the 2010 national census. Across all years and species, there were more males than females with malaria in Tak Province (62.75% males vs 37.25% females) whereas in the general population the proportion of each sex was roughly equal (49.53% males vs 50.47% females). Additionally, 47.41% of cases belonged to the “5–19” age groups in both sexes, whereas in the census, this group comprised only 26.24%, therefore they were overrepresented in the malaria group. There were no major differences in age-sex distribution between the years or between those with *P. falciparum* and *P. vivax*.

### Spatial distribution

**Fig. 7:**
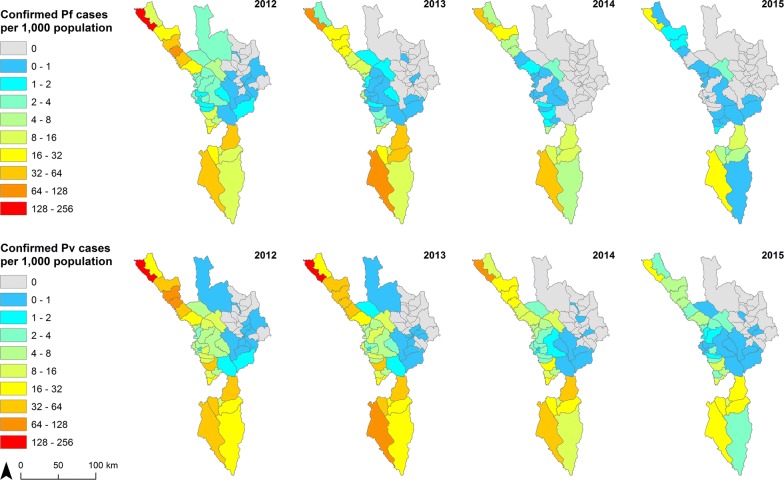
API at sub-district (*tambon*) level in Tak Province, 2012–2015. Mixed infections were added to both *P. falciparum* (Pf) and *P. vivax* (Pv) cases

**Fig. 8:**
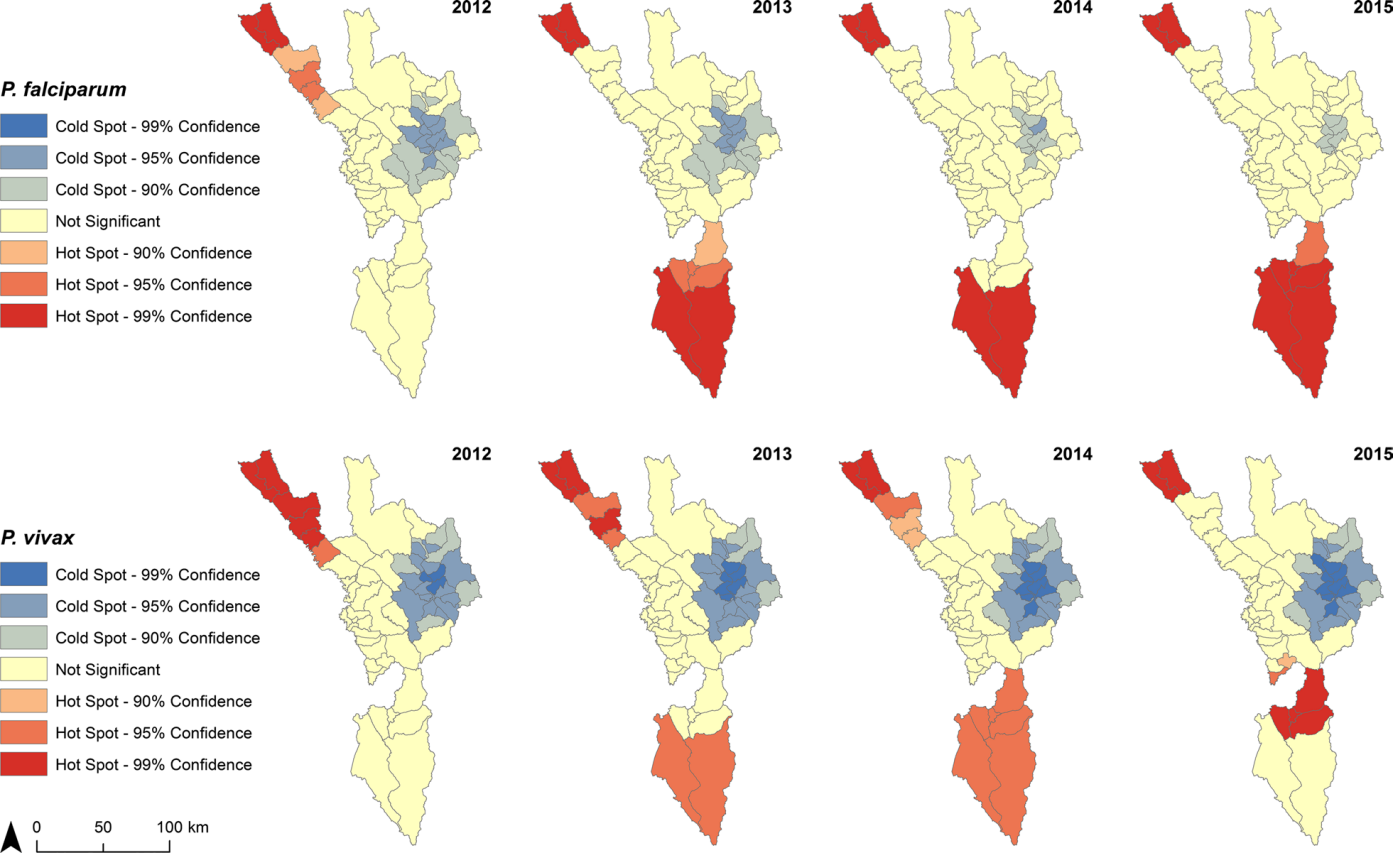
API hot spots by species in Tak Province, 2012–2015. Spatial relationships among features (i.e. sub-district boundaries) were defined by fixed distance band, and calculated with Euclidean (straight-line) distance

There was a decrease in total API in 49 sub-districts and an increase in one from 2012 to 2015. For *P. falciparum,* API decreased in 45 sub-districts. For *P. vivax,* API decreased in 46 sub-districts and an increase in one (Additional file 1: Fig. S3 for confirmed cases). For each measure, the remaining sub-districts (out of the possible 60) were considered malaria-free when there had been no malaria cases in both 2012 and 2015, and therefore no change in API. Sub-districts with the highest API were in the Tha Song Yang and Umphang Districts along the Thai–Myanmar border (Fig. 7). Annual hot spots were mostly in the extreme north and south of the province (Fig. 8). In 2015, the five sub-districts with the highest API (for each of *P. falciparum*, *P. vivax*) were Tha Song Yang (23.09, 31.76), Mokro (10.62, 28.98), Nong Luang (6.48, 19.43), Mae Chan (21.41, 16.38), and Umphang (7.13, 16.37). At least 99% of malaria cases in Tak Province were reported in the five districts that border eastern Myanmar, with most cases reported in Tha Song Yang and Umphang (Table 4).

**Table 4:** Confirmed malaria cases by district in Tak Province, 2012–2015

District	2012		2013		2014		2015		Total cases	
	N	%	N	%	N	%	N	%	N	%
Tha Song Yang	8428	60.83	6389	49.52	2986	47.47	1140	33.95	18,943	52.03
Umphang	2198	15.87	3794	29.40	1931	30.70	1360	40.50	9283	25.50
Phop Phra	1252	9.04	913	7.08	610	9.70	482	14.35	3257	8.95
Mae Sot	1011	7.30	1022	7.92	319	5.07	139	4.14	2491	6.84
Mae Ramat	829	5.98	736	5.70	419	6.66	209	6.22	2193	6.02
Wang Chao	84	0.61	23	0.18	13	0.21	21	0.63	141	0.39
Mueang Tak	34	0.25	20	0.16	10	0.16	6	0.18	70	0.19
Sam Ngao	11	0.08	4	0.03	1	0.02		0.00	16	0.04
Ban Tak	7	0.05	2	0.02	1	0.02	1	0.03	11	0.03
Total	13,854	100.00	12,903	100.00	6290	100.00	3358	100.00	36,405	100.00

Column percentages are presented. There were 131 provincial cases with no district information, not shown

**Fig. 9:**
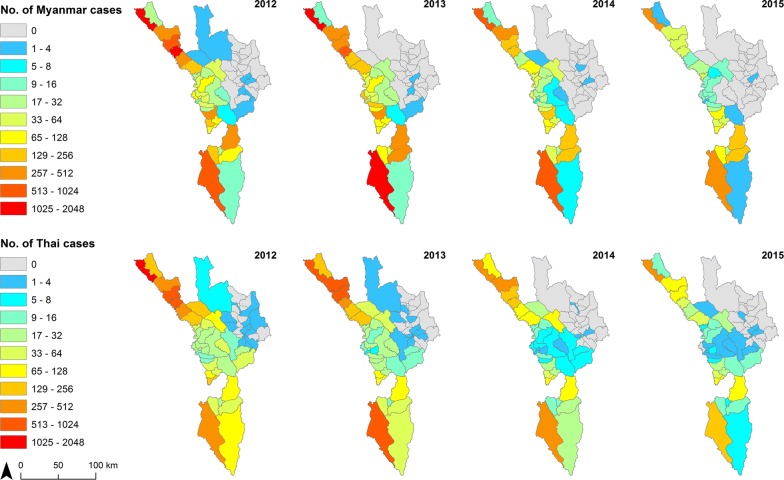
Number of confirmed malaria cases by nationality at sub-district (*tambon*) level in Tak Province, 2012–2015. Top panel maps show total Myanmar cases, while bottom panel maps depict total Thai cases

**Fig. 10:**
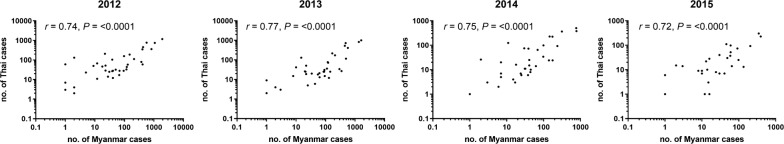
Confirmed Myanmar and Thai malaria cases at sub-district (*tambon*) level in Tak Province, 2012–2015. Correlation test results and axes of scatterplots are in logarithmic scale

**Fig. 11:**
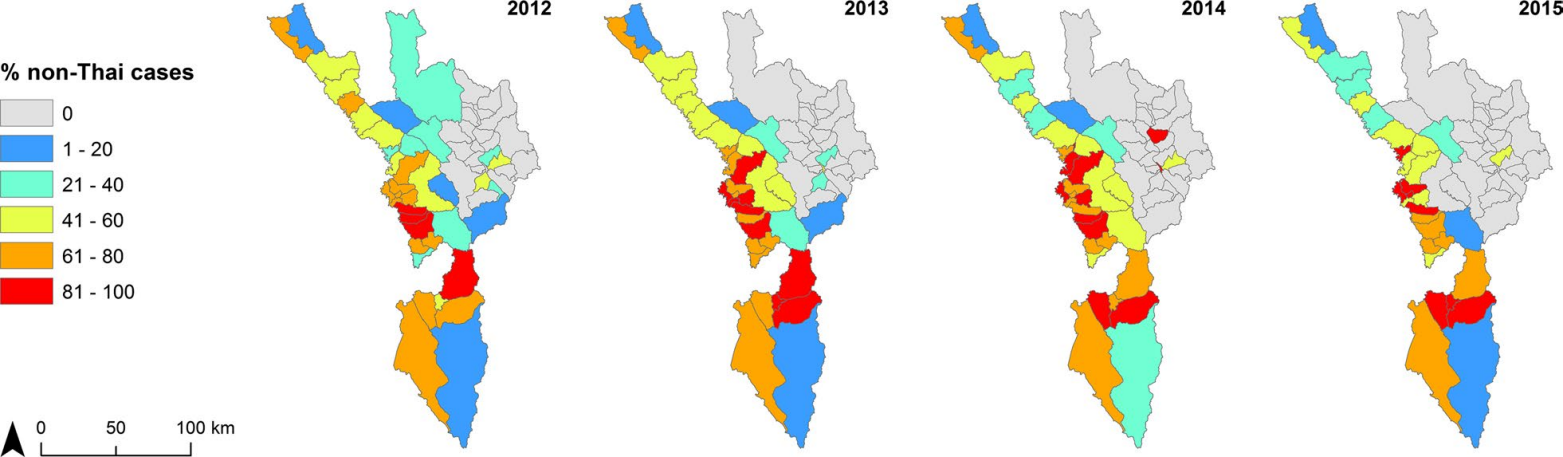
Percent non-Thai malaria (total cases) at sub-district (*tambon*) level in Tak Province, 2012–2015

Figure 9 shows numbers of malaria cases by nationality for Myanmar and Thai (Additional file 1: Fig. S4 for *P. falciparum* plus mixed, Additional file 1: Fig. S5 for *P. vivax* plus mixed). The spatial distribution was similar for both nationalities with a significant correlation in all four years (Fig. 10). When mapped as proportion of cases by nationality, non-Thai cases (93% Myanmar nationals) were more common in the centre of the province in sub-districts nearer to the Myanmar border (Fig. 11).

While many non-Thai cases are seen along the international border with Myanmar, most of them were recorded consistently in specific sub-districts, namely Tha Song Yang (of the same district name), Mae Chan and Mokro (Umphang District) (Fig. 9).

### Forest cover

**Fig. 12:**
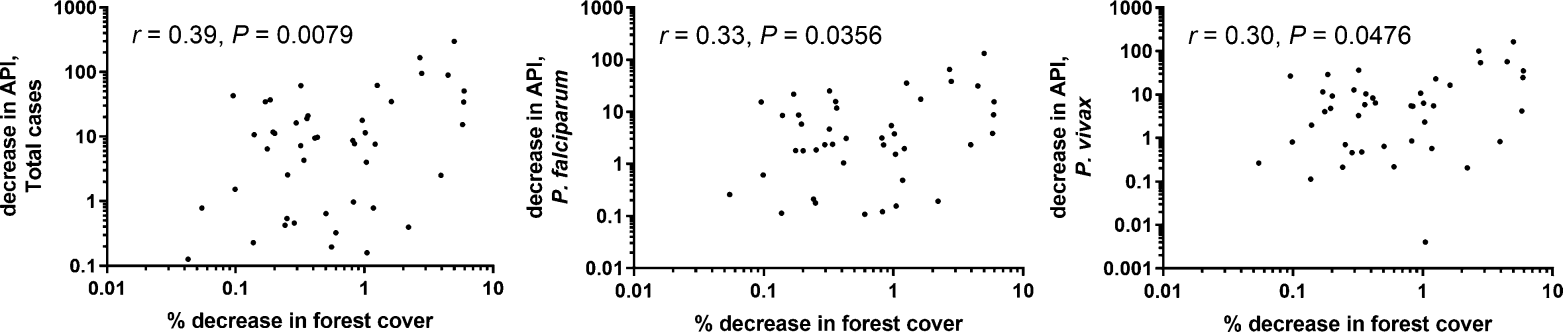
Percent decrease in forest cover and decrease in API at sub-district (*tambon*) level. Correlation test results and axes of scatterplots are in logarithmic scale. Each plot (from left: Total cases, *P. falciparum* plus mixed, *P. vivax* plus mixed) shows the results of Pearson’s correlation test

Satellite images of global forest change available online allowed the derivation of sub-district forest cover expressed as percentage coverage. This was correlated with API. For the whole province, the total percentage forest cover decreased by 1.35%, from 63.42% in 2012 to 62.56% in 2015. The highest percent forest loss was in Tha Song Yang District (4.97%), from 81.14 to 77.10%. The most loss by sub-district being 5.96% in Mae Song and 5.92% in Mae Wa Luang, both in Tha Song Yang District. Percent forest cover decrease by sub-district from 2012 to 2015 did not correlate strongly with change in API over the same period (Fig. 12). At sub-district level, percentage forest cover had a low positive correlation with *P. falciparum*, *P. vivax*, and total cases, within most years (Additional file 1: Fig. S6).

### Climate

Results of correlation of monthly cases per 1000 population and climate variables are shown in Table 5. There were five climate stations in Tak Province. Median temperature (1- and 2-month lags), average temperature (1- and 2-month lags) and average relative humidity (2- and 3-month lags) correlated positively with monthly total, *P. falciparum* and *P. vivax* API. Total rainfall in the same month correlated with API for total cases and *P. vivax,* but not *P. falciparum*. There was no consistent trend in climate variables for each of the five climate stations and no significant correlation between change in climate variables and change in malaria from 2012 to 2015.

**Table 5:** Relationship between climate and malaria incidence in Tak Province, 2012–2015

Variable	Total cases		*P. falciparum*		*P. vivax*	
	*r*	*P*	*r*	*P*	*r*	*P*
Rainfall (cm)						
Total, no lag	*0.33*	*0.026*	0.27	0.066	*0.36*	*0.015*
Total, 1-month lag	0.09	0.564	0.04	0.813	0.12	0.438
Total, 2-month lag	− 0.11	0.463	− 0.13	0.384	− 0.10	0.513
Total, 3-month lag	− 0.15	0.322	− 0.14	0.355	− 0.16	0.309
Relative humidity (%)						
Minimum	0.19	0.195	0.13	0.382	0.23	0.115
Maximum	0.28	0.056	0.22	0.136	*0.31*	*0.033*
Range (Max − Min)	− 0.03	0.832	0.01	0.962	− 0.07	0.663
Average, no lag	0.26	0.076	0.19	0.197	*0.30*	*0.038*
Average, 1-month lag	− 0.02	0.871	− 0.06	0.677	0.00	0.981
Average, 2-month lag	− *0.35*	*0.019*	− *0.34*	*0.021*	− *0.34*	*0.020*
Average, 3-month lag	− *0.47*	*0.001*	− *0.41*	*0.005*	− *0.51*	*<* *0.001*
Temperature (°C)						
Minimum	0.14	0.330	0.08	0.580	0.18	0.225
Maximum	0.09	0.549	0.06	0.663	0.10	0.503
Range (Max − Min)	− 0.14	0.353	− 0.06	0.705	− 0.19	0.201
Median, no lag	0.06	0.670	0.03	0.838	0.08	0.589
Median, 1-month lag	*0.38*	*0.008*	*0.33*	*0.024*	*0.41*	*0.004*
Median, 2-month lag	*0.47*	*0.001*	*0.39*	*0.007*	*0.52*	*<* *0.001*
Median, 3-month lag	0.28	0.062	0.20	0.199	*0.33*	*0.025*
Average, no lag	0.07	0.619	0.04	0.808	0.09	0.528
Average, 1-month lag	*0.38*	*0.008*	*0.33*	*0.024*	*0.41*	*0.004*
Average, 2-month lag	*0.47*	*0.001*	*0.39*	*0.008*	*0.52*	*<* *0.001*
Average, 3-month lag	0.28	0.061	0.20	0.196	*0.33*	*0.025*

Climate measurements and parasite incidence were aggregated by month. *r* and P-values in italic text were those that were significant at alpha level of 0.05

## Discussion

From 2012 to 2015, Tak Province has experienced a substantial and consistent decline of both *P. falciparum* and *P. vivax* malaria with a decline in *P. falciparum*-to-*P. vivax* ratio over the same period. Around three quarters of the cases and areas with highest risk (indicated by API) were consistently in clusters of a few sub-districts in two out of the nine districts (Tha Song Yang and Umphang) at the northern and southern extremities of the province by the Thai–Myanmar border. There was some correlation with the percent decrease in forest cover and decline in API at sub-district level. This geographical pattern was similar for both species.

Although climate correlated reasonably well with API, there was no clear change in seasonal climate pattern over the period of the study. This suggests that change in climate was not responsible for the decrease in malaria. The relationship between trends in climate and malaria is not straightforward in that there is an optimal ecological niche of climate within which mosquito breeding and malaria transmission are more efficient [33]. In Thailand, there are seven Anopheles species known to transmit malaria, which have different environmental optima and geographic distributions and it is not clearly known to what degree they contribute to transmission in Tak Province [34]. Because the correlation was weak, the change in forest cover is unlikely to have been the main driver overall for the reduction in malaria. It is possible that small changes in forest cover may have a greater contribution in small foci where forest transmission is occurring but it was not possible to study this, as suitable detailed data was not available. More likely, it is a result of the Thai MOPH’s ongoing elimination efforts in the area with high coverage of long-lasting insecticidal nets, good access to diagnosis and treatment via malaria posts and health promoting hospitals, active case detection and village health volunteers existing with vector control and facilitating referral of symptomatic individuals [2]. However, it was not possible to assess this as suitable data on interventions were not available. This decline in cases mirrors that of what is seen elsewhere in Thailand, and in subsequent years, with overall strong progress towards the national goal of elimination [2]. Also unique to Tak Province is the presence of Shoklo Malaria Research Unit (SMRU) in Mae Sot District, which has been providing early diagnosis and treatment for malaria since 1986 [35]. SMRU operates at both sides of the Thai–Myanmar border, and reported 17,446 malaria cases between 2012 and 2015 (Additional file 1: Table S1 for annual reported cases by species) at sites in Tak Province with an 85.64% decline in reported malaria cases from 2012 to 2015. There was a similar decline in cases reported by the Thai MOPH at 75.78% in the same period. SMRU sites in Kayin State, Myanmar also saw a decline in malaria incidence, as brought about by recent gains in elimination efforts in Myanmar [36]. Due to migration of people between the two areas, the decline in reported cases on the Thai side may also be partly due to the decline on the other side of the border. Lack of functioning malaria posts in Tha Song Yang (2014) and Umphang (2015) Districts (source: SMRU unpublished reports) may have contributed to increased transmission in the areas identified as hotspots in this analysis.

A strength of this study is that it combined detailed data from all sources reporting to the MOPH in Thailand together [37, 38], with population data from the same area. This allowed examination of which groups of people had the highest rates of malaria in each major species. Similar to other studies in the region [39–41] and elsewhere [42], around two-thirds were male, particularly in young adults, possibly due to increased occupational risk and forest exposure in this group. More than half the cases were of Myanmar nationality. Data were not available to indicate whether they were short-term or long-term migrants, but this study mapped cases by place of residence where this information was available (i.e. which sub-district a patient confirmed to have malaria was reported to be from). When cases were mapped by nationality, those of Myanmar nationality were clustered in a few sub-districts in the centre of the province along the country border.

The study had several important limitations. Although the data was of high quality, there was some missing information, e.g. 2.40% of cases had unknown malaria species, 13.88% of patients had no age recorded, 4.08% had no nationality recorded, and 1.30% had missing sub-district codes (Additional file 1: Table S2 for yearly percentages, and 4-year average). The completeness of data for each field did not consistently increase over the period of the study. The study utilized data obtained mostly from passive case detection with a few from active case detection (Additional file 1: Table S3 for an annual breakdown of confirmed malaria cases by detection method), but it was not clear which applied to each case. There are other sources of surveillance data that are not fully captured in the MOPH database, including those from the military and non-governmental organizations [43]. PCR is not used for routine surveillance in Thailand and there was no available data from the government on asymptomatic infections, which could render asymptomatic cases to be undetected, as some research studies have revealed in both high transmission [44, 45] and low transmission [46, 47] settings at the Thai–Myanmar border. Data were not included from SMRU, and these additional case data could be substantial enough to affect the spatial and temporal patterns of disease found in this study. However, it is not clear to what degree cases recorded by SMRU overlap with the dataset used in this study due to, e.g. presentation of cases to more than one organization within the same malaria episode, and there is no system to effectively integrate the two databases accounting for this potential overlap. Perhaps more importantly, many of these cases are short-term migrants who are likely to have been infected in Myanmar, there being a porous border between the two countries in this area. Including cases from SMRU should thus have had little impact on the results as the analysis was based on place of residence being in Tak Province. Other data that were not available for the analysis were numbers of people tested, or detailed data on interventions including bed net distribution or treatment, which could have been used to confirm the downward trend in cases being due to successful elimination efforts. Finally, the data used to map forest cover had limitations in that there was no information on forest gain from 2013 to 2015 (stable forest gain was assumed during this period), only forest loss [26].

It would be informative to explore further about possible causes of the spatial pattern of disease. For example, delays in seeking treatment [48], importation or spread through human movement [49, 50], travel into the forest [51], locations of particular occupational risk groups, or the role of undiagnosed cases [52] and parasite genetics [53]. This would require additional surveys to collect this information. Another possible future analysis would be to use satellite remote sensing data as a higher resolution source of information on climate to examine the variation across space. More detailed mapping of the distribution of cases to assess the degree of clustering would also be helpful but this would require village level data on place of residence, which was not available for this study. This is particularly important as further progress is made towards elimination requiring targeting of these clusters to reach zero. It would also be informative to include data from neighbouring areas of Myanmar [54] to assess risk at either side of the border. Given the cross-border nature of the study setting [55], and how this could affect malaria transmission, there is an opportunity to advocate for a regional data system that would allow for a combined analysis of trends for both Tak Province in Thailand and Kayin State in Myanmar.

## Conclusions

There has been a large decline in reported clinical malaria from 2012 to 2015 in Tak Province. Climate and forest correlated with malaria incidence rates but did not account for this decrease. Ongoing elimination interventions on one or both sides of the border are more likely to have been the cause but it was not possible to assess this due to a lack of suitable data. Two main hot spot areas were identified along the Thai–Myanmar border that should be studied in more detail so they can be targeted for elimination activities.

## Additional file

**Additional file 1.** Additional figures and tables.

## Data Availability

The malaria surveillance dataset is generated and owned by the Department of Disease Control, Ministry of Public Health (MOPH), Thailand, but are available on reasonable request.

## Abbreviations

API: annual parasite incidence
BOE: Bureau of Epidemiology
BVBD: Bureau of Vector-borne Diseases
MOPH: Ministry of Public Health, Thailand
PDR: Lao People’s Democratic Republic
RDT: rapid diagnostic test
SMRU: Shoklo Malaria Research Unit
