# Supplementary material for: Spatiotemporal epidemiology, environmental correlates, and demography of malaria in Tak Province, Thailand (2012–2015)
Source: Malar J. 2019 Jul 16;18:240. doi: 10.1186/s12936-019-2871-2 (PMC6636027; doi:10.1186/s12936-019-2871-2)
Supplement: Supplementary file 1 — Additional file 1. Additional figures and tables. [file 12936_2019_2871_MOESM1_ESM.docx]

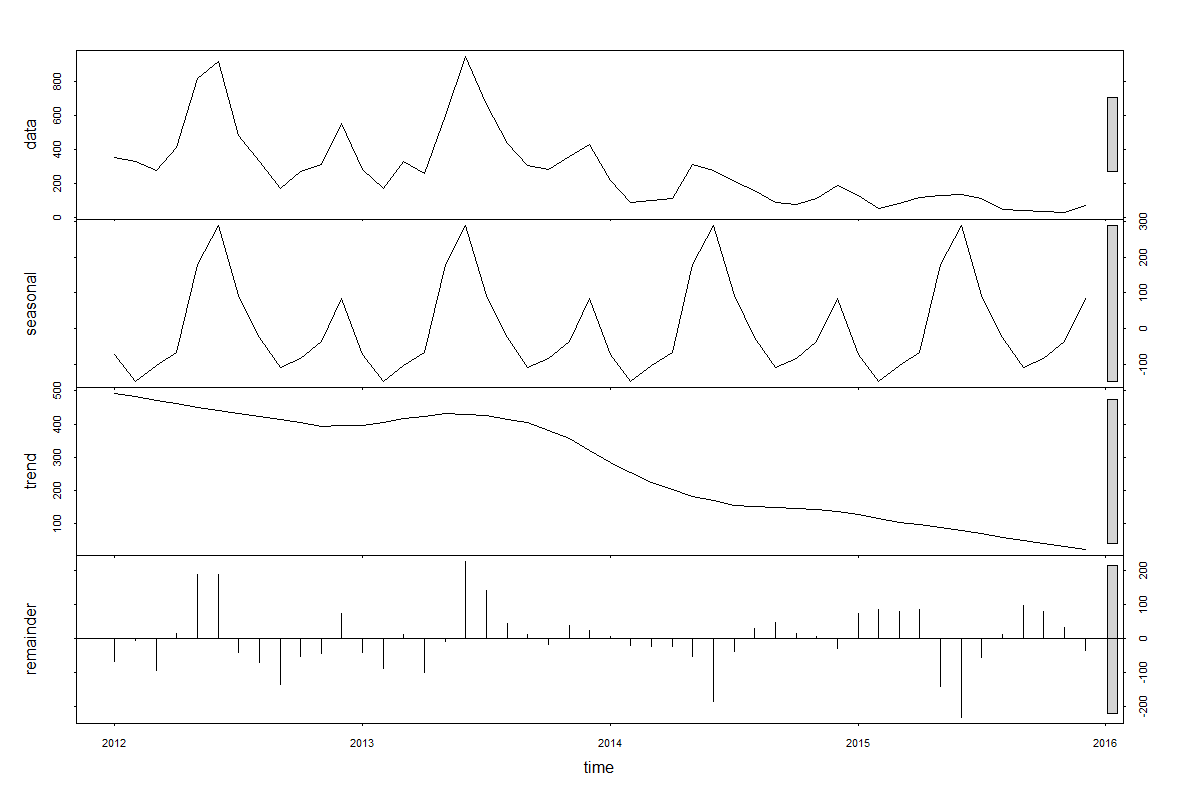
Fig. S1 Time series decomposition of total confirmed *P. falciparum* malaria cases in Tak Province, 2012-2015. Top plot is decomposed into seasonal, trend and irregular components. The grey bar on the right indicates the relative magnitude of each of the decomposed components (length of bar = relative magnitude).


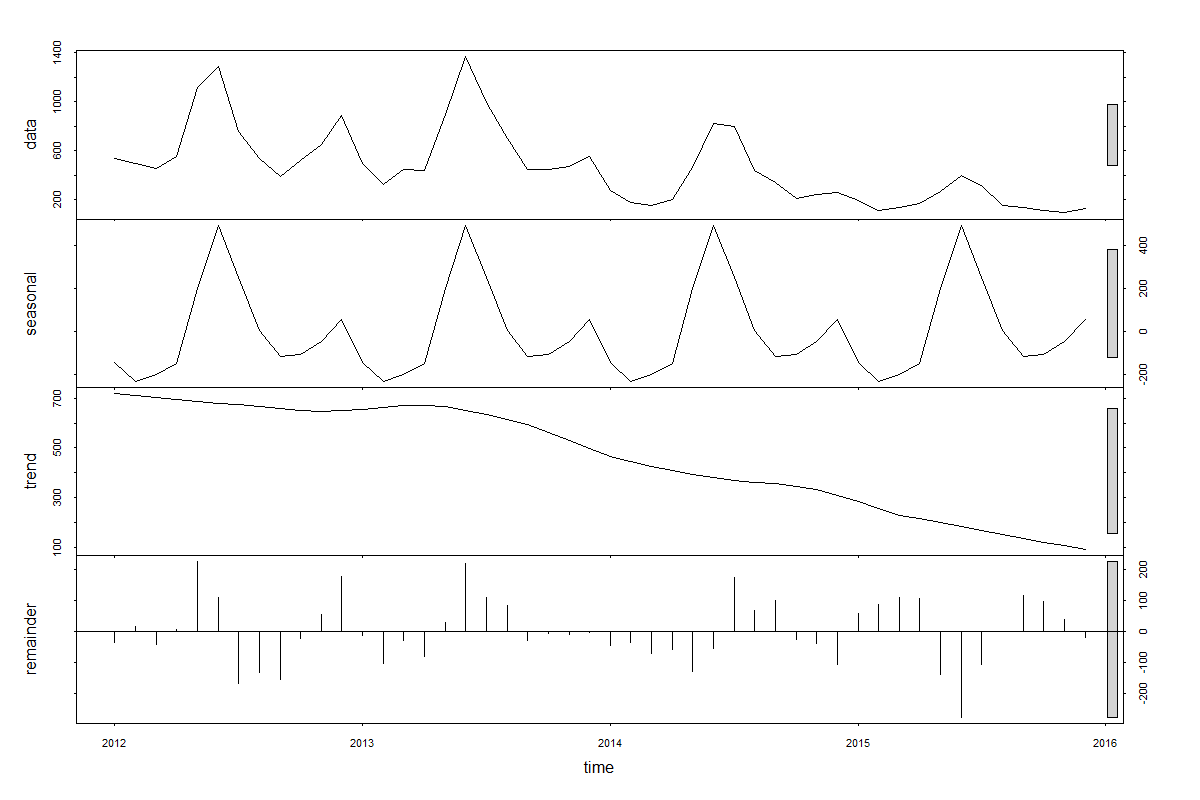


Fig. S2 Time series decomposition of total confirmed *P. vivax* malaria cases in Tak Province, 2012-2015. Top plot is decomposed into seasonal, trend and irregular components. The grey bar on the right indicates the relative magnitude of each of the decomposed components (length of bar = relative magnitude).


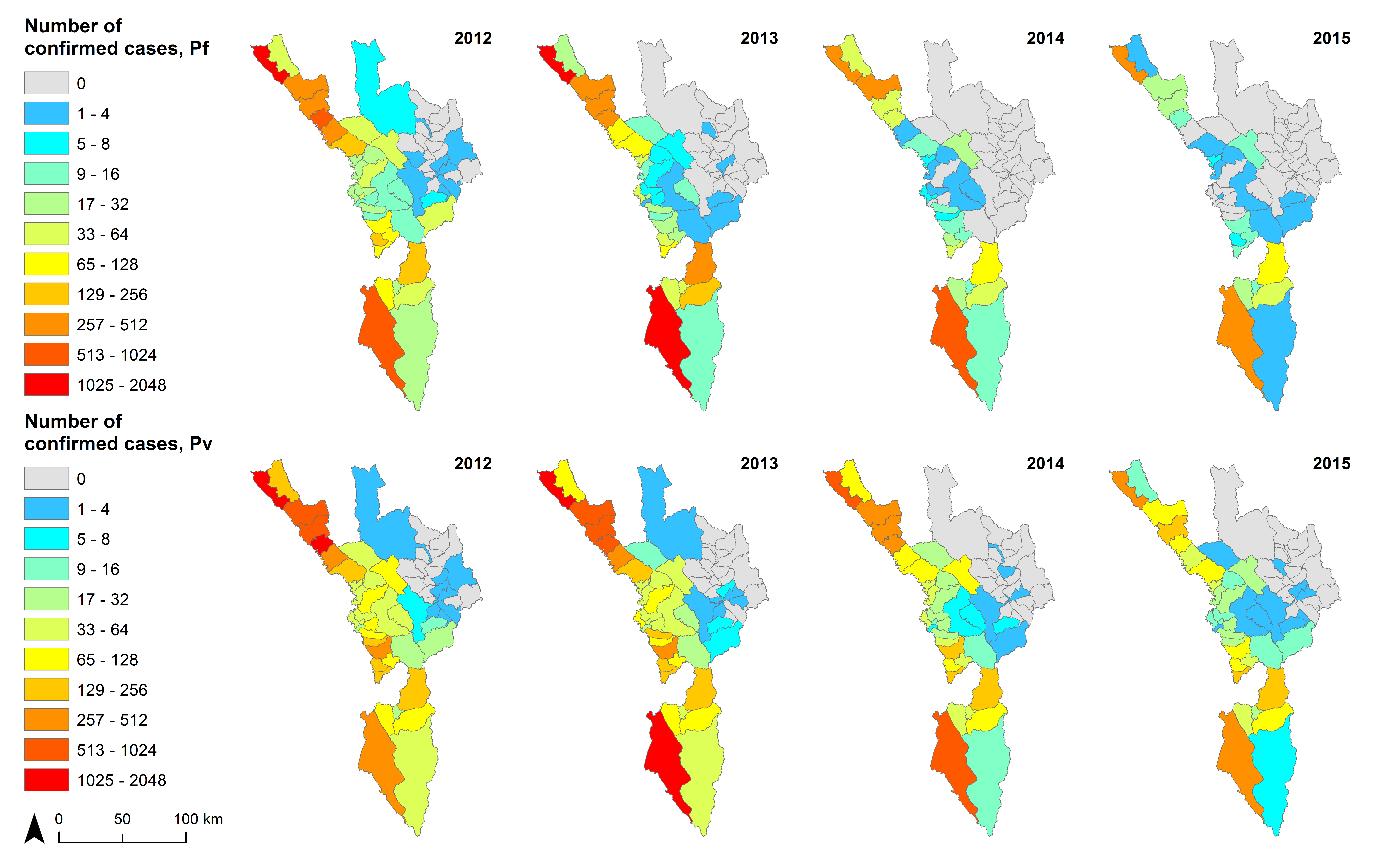


Fig. S3 Number of confirmed malaria cases (top panel: *P. falciparum* plus mixed infections, lower panel: *P. vivax* plus mixed infections) at subdistrict (*tambon*) level in Tak Province, 2012-2015.


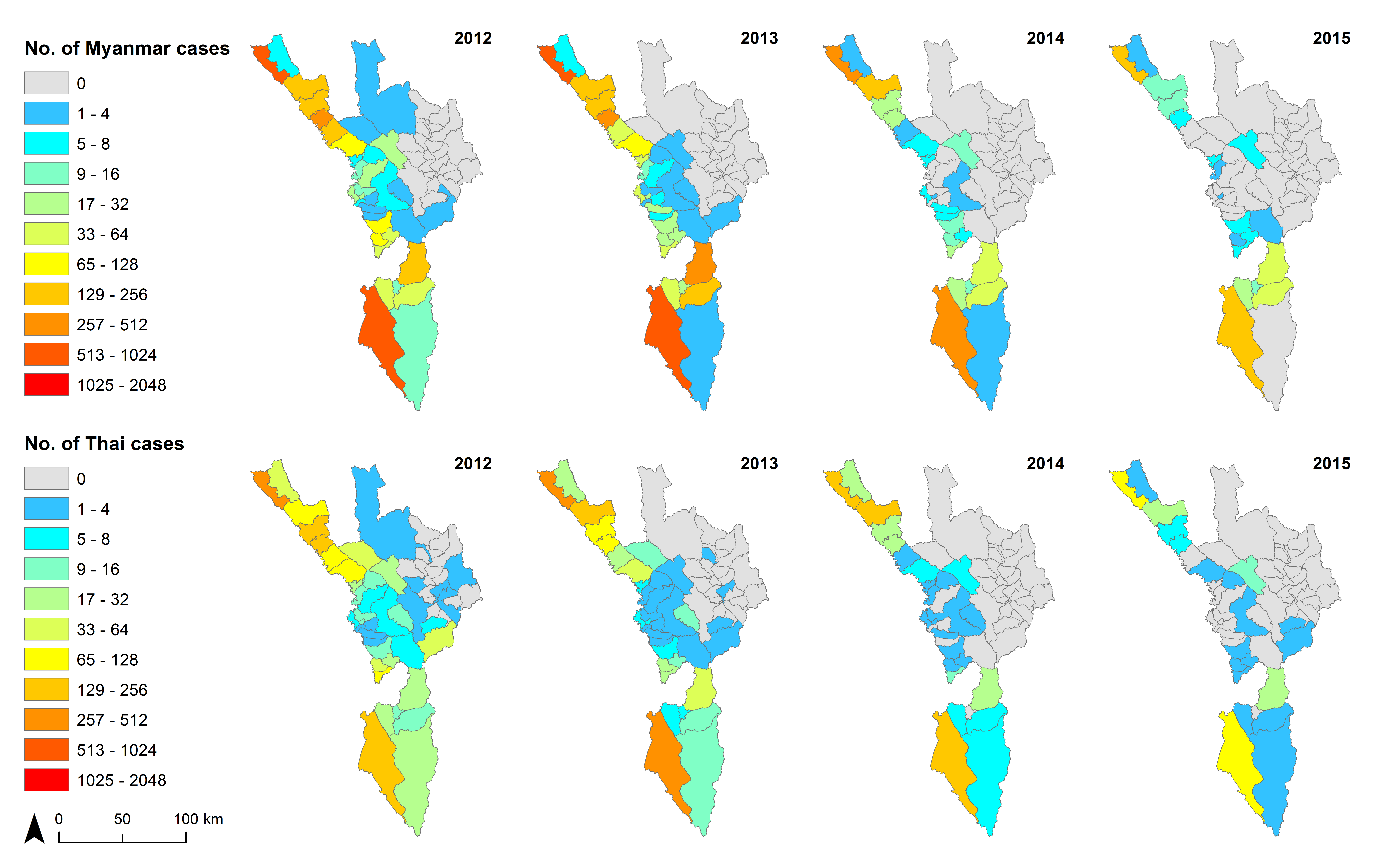


Fig. S4 Number of confirmed malaria cases (*P. falciparum* plus mixed infections) by nationality at subdistrict (*tambon*) level in Tak Province, 2012-2015. Top panel maps show Myanmar cases, while bottom panel maps depict Thai cases.


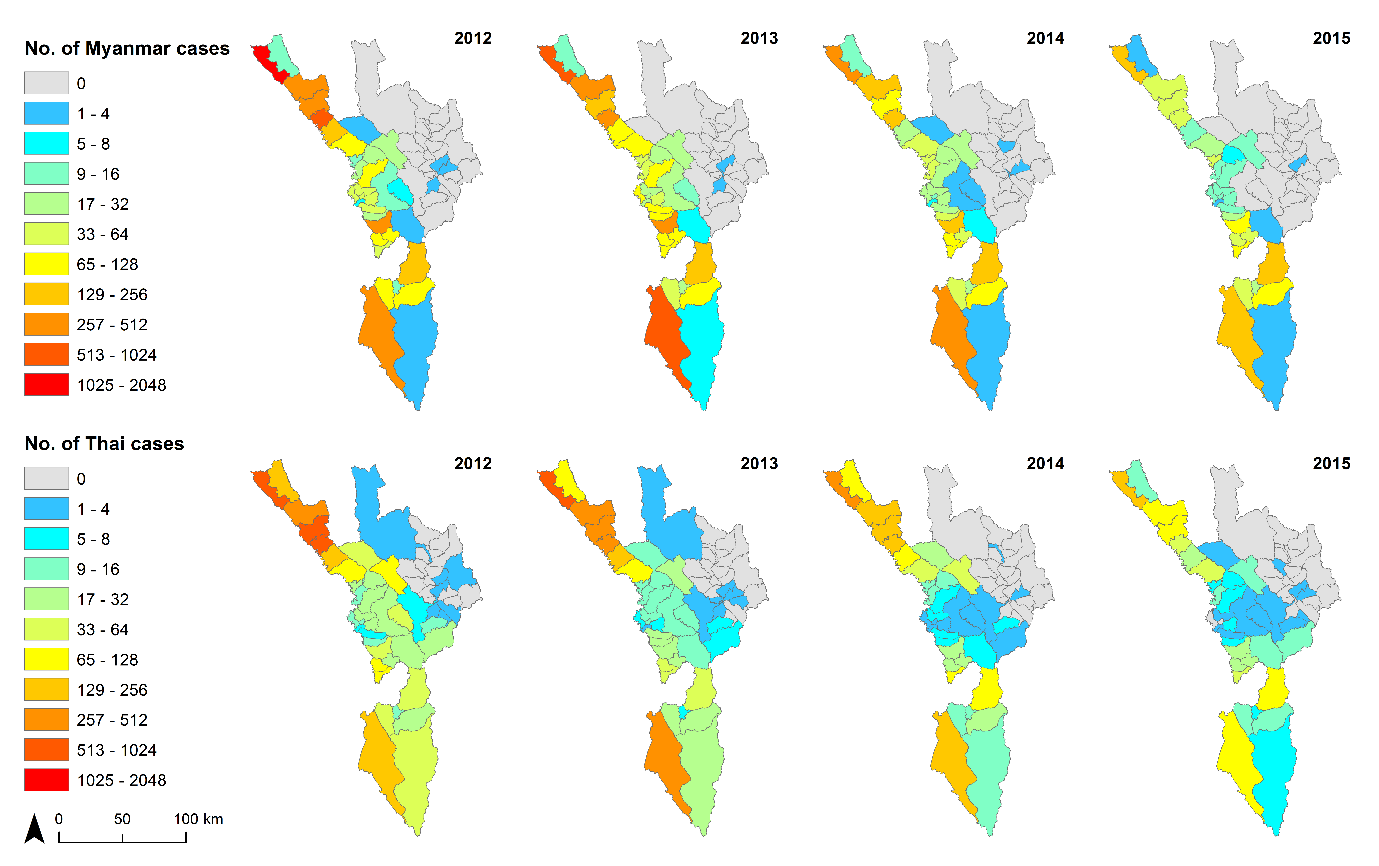


Fig. S5 Number of confirmed malaria cases (*P. vivax* plus mixed infections) by nationality at subdistrict (*tambon*) level in Tak Province, 2012-2015. Top panel maps show Myanmar cases, while bottom panel maps depict Thai cases.


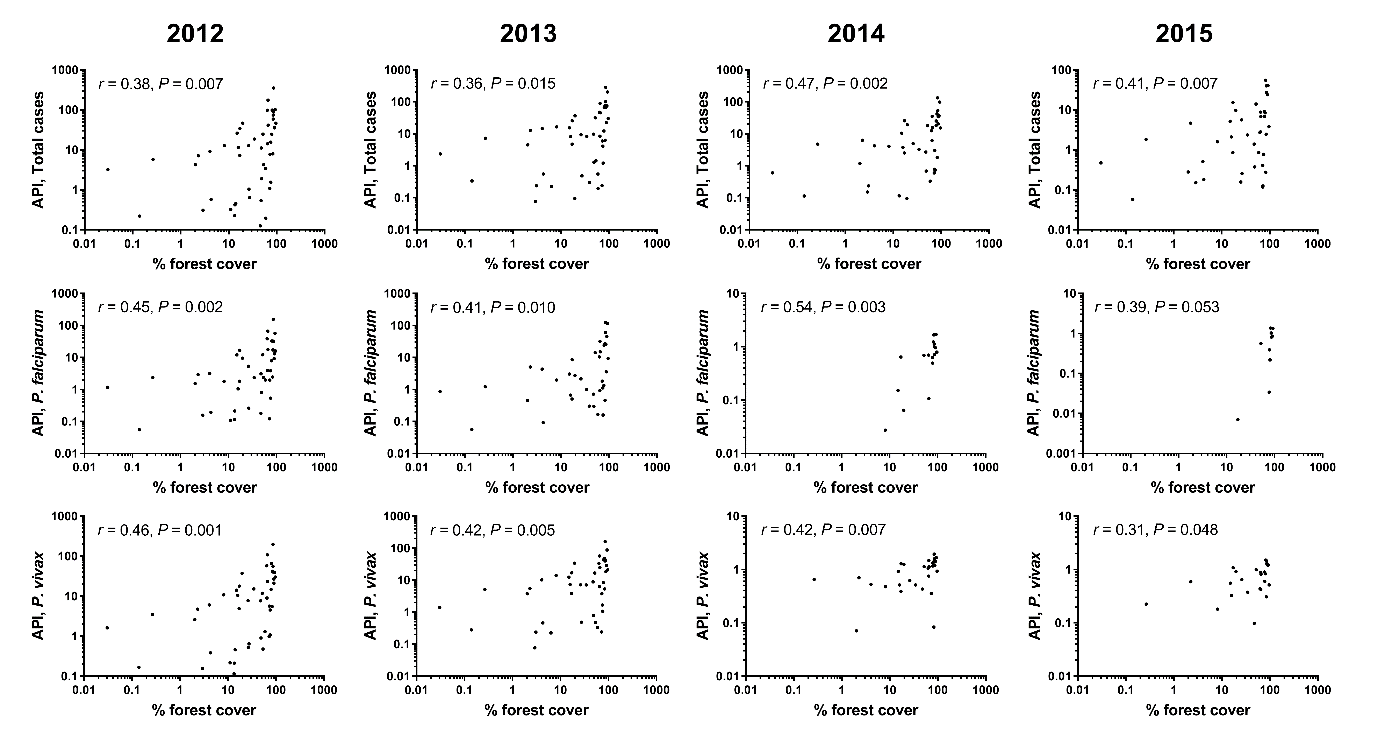


Fig. S6 Scatterplots (logarithmic scale) of percent forest cover and API at subdistrict (*tambon*) level in Tak Province, 2012-2015. Rows represent species of malaria (mixed infections were added to both *P. falciparum* and *P.* *vivax* cases), while columns represent years of surveillance. Each plot shows the results of Pearson’s correlation test.

Table S1 Number of confirmed malaria cases in SMRU for Tak Province, 2012-2015.

| Year | *P. falciparum* | *P. vivax* | *P. malariae* | *P. ovale* | Total |
| --- | --- | --- | --- | --- | --- |
| 2012 | 3,820 | 4,768 | 234 | 1 | 8,823 |
| 2013 | 1,762 | 3,558 | 11 | 0 | 5,331 |
| 2014 | 245 | 1,770 | 9 | 1 | 2,025 |
| 2015 | 94 | 1,169 | 4 | 0 | 1,267 |
| Total | 5,921 | 11,265 | 258 | 2 | 17,446 |

Table S2 Summary of missing information in the malaria dataset for Tak Province, 2012-2015.

| Year | Age | | Nationality | | Species | | Subdistrict code | |
| --- | --- | --- | --- | --- | --- | --- | --- | --- |
|  | N | % | N | % | N | % | N | % |
| 2012 | 2,038 | 14.70 | 606 | 4.37 | 353 | 2.55 | 98 | 0.71 |
| 2013 | 1,727 | 13.38 | 470 | 3.64 | 253 | 1.96 | 197 | 1.53 |
| 2014 | 712 | 11.10 | 233 | 3.63 | 101 | 1.58 | 176 | 2.74 |
| 2015 | 595 | 17.72 | 180 | 5.36 | 171 | 5.09 | 4 | 0.12 |
| Overall | 5,072 | 13.88 | 1,489 | 4.08 | 878 | 2.40 | 475 | 1.30 |
| Average | 1,268 | 14.23 | 372.25 | 4.25 | 219.50 | 2.79 | 118.75 | 1.27 |

Table S3 Malaria cases by detection method in Tak Province, 2012-2015.

| Year | Passive case  detection | | Active case detection | | Follow-up | | Total | |
| --- | --- | --- | --- | --- | --- | --- | --- | --- |
|  | N | % | N | % | N | % | N | % |
| 2012 | 13,074 | 94.31 | 543 | 3.92 | 246 | 1.77 | 13,863 | 100.00 |
| 2013 | 12,139 | 94.08 | 539 | 4.18 | 225 | 1.74 | 12,903 | 100.00 |
| 2014 | 5,918 | 92.30 | 349 | 5.44 | 145 | 2.26 | 6,412 | 100.00 |
| 2015 | 3,075 | 91.57 | 213 | 6.34 | 70 | 2.08 | 3,358 | 100.00 |
| Total | 34,206 | 93.62 | 1,644 | 4.50 | 686 | 1.88 | 36,536 | 100.00 |

Note: Row percentages are presented. “Follow-up” refers to cases on follow-up visits after treatment, which are likely to be duplicated from passive and active case detection methods.
